# Mechanistic insights into the acetyl-CoA recognition by SLC33A1

**DOI:** 10.1038/s41421-025-00793-1

**Published:** 2025-04-10

**Authors:** Dong Zhou, Nanhao Chen, Shitang Huang, Chen Song, Zhe Zhang

**Affiliations:** 1 State Key Laboratory of Membrane Biology, School of Life Sciences, Peking University, Beijing, China; 2 Center for Life Sciences, Academy for Advanced Interdisciplinary Studies, Peking University, Beijing, China; 3 Center for Quantitative Biology, Academy for Advanced Interdisciplinary Studies, Peking University, Beijing, China; 4 Isotope Laboratory, School of Life Sciences, Peking University, Beijing, China

**Keywords:** Cryoelectron microscopy, Endoplasmic reticulum

Dear Editor,

Acetyl-coenzyme A (acetyl-CoA) is a pivotal metabolic intermediate that plays dual roles: it is both a product of numerous catabolic reactions and a precursor for a multitude of anabolic processes. In addition to these metabolic functions, acetyl-CoA acts as a donor for the acetylation modification of various proteins and glycolipids, which is crucial for regulating their activity and function^1^. Acetyl-CoA is widely present throughout various cellular compartments, such as the cytoplasm, nucleus, mitochondria, endoplasmic reticulum (ER), and peroxisomes. Maintaining the balance of acetyl-CoA in these organelles is vital for cellular metabolism and protein homeostasis^1,2^.

Acetyl-CoA, a large and charged molecule (Fig. 1a), is unable to freely diffuse across cellular membrane. Thus, it requires specific transporter proteins to facilitate its transmembrane movement, either directly or after conversion into other compounds like citrate. To date, SLC33A1, also known as AT-1, is the only identified acetyl-CoA transporter within the cell^3^. This protein is localized to the ER membrane and is responsible for transporting acetyl-CoA from the cytosol to the ER lumen^4^. Acetyl-CoA in the ER primarily serves to acetylate the lysine residues of secreted and membrane proteins, as well as the terminal sialic acid residues of gangliosides^2,3^. Similar to glycosylation, proper acetylation of secretory proteins is a critical step in assisting protein folding and maturation^4^. Additionally, the acetylation status of certain ER proteins is crucial for modulating the ER-specific autophagic process, which is essential for the clearance of toxic protein aggregates^5,6^. On another note, the acetylation of gangliosides exerts multiple effects, including the modification of their antigenic properties and the inhibition of apoptosis^7^. Notably, disruptions to SLC33A1, whether due to insufficient or excessive activity, can lead to various diseases, especially those affecting the nervous system^2^. For instance, the S113R mutation in SLC33A1, a malfunctioning variant, is known to cause autosomal-dominant spastic paraplegia (SPG42) in human patients^8^. Heterozygous mice harboring this mutation exhibit neurodegeneration and are more susceptible to infections, inflammation, and cancer^9^. Conversely, the overexpression or upregulation of SLC33A1 has been implicated in sporadic amyotrophic lateral sclerosis^10^ and is associated with phenotypes resembling autism and progeria^11,12^. Although SLC33A1 plays a key role in regulating acetyl-CoA homeostasis between the cytosol and the ER^13^, our understanding of its function has been constrained by the absence of a high-resolution structure. Here, we report the cryo-electron microscopy (cryo-EM) structure of human SLC33A1 in complex with acetyl-CoA. Along with functional analyses, we have revealed the substrate recognition mechanism of this key acetyl-CoA transporter.

**Fig. 1:**
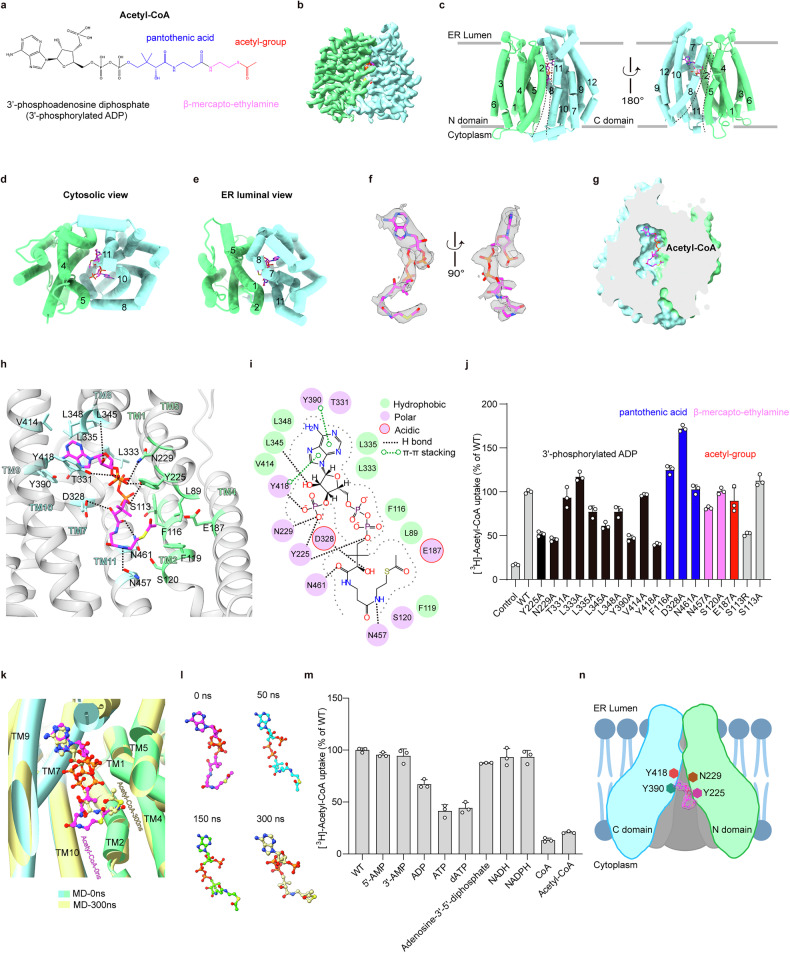
Molecular mechanism of acetyl-CoA recognition by SLC33A1. **a** Schematic diagram of acetyl-CoA. **b** Cryo-EM map of the SLC33A1/acetyl-CoA complex. **c**–**e** Overall structure of acetyl-CoA-bound SLC33A1 in the cytoplasm-facing conformation viewed from the membrane plane (**c**), the cytosolic side (**d**), or the ER luminal side (**e**). TM1–6 (N domain) are colored in light green, while TM7–12 (C domain) are colored in pale turquoise. Acetyl-CoA is shown in magenta. **f** Cryo-EM density of acetyl-CoA. **g** Surface representation of the acetyl-CoA-binding site in SLC33A1. **h**, **i** Details of the interaction between SLC33A1 and acetyl-CoA. The dashed lines represent hydrogen bonds, salt bridges, or electrostatic interactions (< 4.0 Å). The residues involved in van der Waals and hydrophobic interactions (< 4.0 Å) are shown with side chains. Ligand interactions are analyzed using ChimeraX (**h**) and MOE (**i**) software, respectively. **j** The [^3^H]-acetyl-CoA uptake activities of wild-type (WT) SLC33A1 and its mutants. The results are normalized to the activity of WT SLC33A1. All experiments were done in triplicate (*n* = 3, means ± SD). One hundred percent transport activity was defined as the average [^3^H]-acetyl-CoA signal transported by WT SLC33A1. The histograms for residues involved in interacting with different moieties of acetyl-CoA are color-coded and indicated accordingly. The gray columns represent the control, WT, and disease-related mutants. **k** Overlay comparison of the poses of acetyl-CoA between our structural model and the 300 ns MD simulation result. Acetyl-CoA is colored in magenta and light khaki, respectively. **l** Conformations of acetyl-CoA at various time points during the 300 ns MD simulation. Acetyl-CoA is shown as sticks. **m** Inhibitory effect of different compounds on the [^3^H]-acetyl-CoA uptake activities of SLC33A1. All the molecules were tested at a concentration of 250 μM. The results were normalized to the activity of the control experiment in which no compounds were added. All experiments were done in triplicate (*n* = 3, means ± SD). **n** A schematic illustration of the acetyl-CoA recognition mechanism of SLC33A1, with the four key residues (Y225, N229, Y390, and Y418) involved in substrate recognition highlighted.

We purified the human SLC33A1 protein and reconstituted it into saposin-based nanoparticles. Subsequently, we resolved the cryo-EM structure of the SLC33A1/acetyl-CoA complex to a resolution of 3.5 Å (Fig. 1b; Supplementary Fig. S1 and Table S1). In this structure, SLC33A1 exhibits a typical fold characteristic of the major facilitator superfamily (MFS), adopting a cytoplasm-facing conformation (Fig. 1c). Specifically, the two lobes of the protein, TM1–6 (N domain) and TM7–12 (C domain), are oriented towards the cytoplasm between TM4–5 and TM10–11 (Fig. 1d), aligning with the AlphaFold prediction^14^ (Supplementary Fig. S2a). Of note, the two halves of SLC33A1 open to the membrane’s edge between TM5 and TM8 near the cytosol but are securely closed on the opposite side, between TM2 and TM11. Additionally, the linker region connecting TM6 and TM7 is positioned externally to TM2 and TM11 (Fig. 1c). This configuration strongly suggests that the substrates would likely access the central cavity through an intracellular portal framed by TM4, TM5, TM8, TM10, and TM11 (Fig. 1d). On the ER side, the luminal gate is predominantly sealed by the interaction of TM1–2 and TM7–8. Furthermore, TM5 and TM11 also play a role in reinforcing the gate’s stability by providing additional support on the flanks (Fig. 1e).

A distinct EM density was observed within the central cavity of SLC33A1 (Fig. 1f, g), positioned near the luminal side and extending into the gap between transmembrane helices TM7–10 (Fig. 1g, h). This pronounced density closely matches the terminal 3′-phosphoadenosine diphosphate (3′-phosphorylated ADP) component of acetyl-CoA (Fig. 1a, f). Despite the somewhat weaker EM density for the remaining portion of acetyl-CoA (Supplementary Fig. S2b), we were able to approximate its general location (Fig. 1f). In our structural model, the 3′-phosphorylated ADP moiety of acetyl-CoA primarily interacts with TM7–10 of the C domain, with additional assistance from TM1, TM2, and TM5 of the N domain (Fig. 1h, i). The flexible pantothenic acid and β-mercaptoethylamine groups of CoA extend into the central region of the transporter, adopting an overall coiled conformation. The sharp rotation facilitated by the two amide bonds positions the terminal β-mercaptoethylamine and acetyl groups parallel to the membrane plane (Fig. 1h, i). Notably, these pliable components of acetyl-CoA mainly interact with the closed lateral side of the transporter, engaging in contacts with TM2 and TM11 (Fig. 1h, i). In particular, the adenine group is nestled within a hydrophobic pocket formed by residues Thr331, Leu335, Leu345, Leu348, Tyr390, Val414, and Tyr418. Among them, the two tyrosine residues, Tyr390 and Tyr418, encase the adenine ring through π–π interactions (Fig. 1h, i; Supplementary Fig. S2c). The ribose and phosphate groups engage in extensive polar interactions with surrounding residues. For instance, the 2′-hydroxyl group of ribose forms a hydrogen bond with the backbone carbonyl of Leu345, the 3′-phosphate interacts with Tyr225, Asn229, and Tyr418, and the 5′-pyrophosphate is cradled by Phe116 and Tyr225 (Fig. 1h, i; Supplementary Fig. S2c). Moreover, the tail part of acetyl-CoA, including pantothenic acid, β-mercaptoethylamine, and acetyl groups, also align with several residues through hydrophilic or van der Waals interactions along their extended direction, including Asp328, Asn461, Asn457, Ser120, Phe119, Leu89, and Glu187 (Fig. 1h, i; Supplementary Fig. S2c).

To verify the acetyl-CoA-binding site, we individually mutated the surrounding residues observed in our structure to alanine and examined the impact of these mutations on the substrate transport activity of SLC33A1. After expressing the wild-type and mutated variants of the protein in HEK293T cells, we isolated the ER microsomes and quantified their acetyl-CoA transport activity using a radiolabeled substrate ([^3^H]-acetyl-CoA) (Fig. 1j; Supplementary Fig. S3). Our results revealed that mutations Y225A, N229A, Y390A, and Y418A significantly impaired the transport activity of SLC33A1, highlighting the importance of the polar and π–π stacking interactions with the 3′-phosphorylated ADP moiety for the recognition of acetyl-CoA by SLC33A1 (Fig. 1h, i). In contrast, mutations of the hydrophobic residues involved in 3′-phosphorylated ADP binding, as well as mutations of other residues that contact the remaining part of acetyl-CoA, only had a modest effect on substrate transport (Fig. 1j). This suggests that these interactions are less critical to substrate recognition. Importantly, our molecular dynamics (MD) simulations further revealed that the binding of the 3′-phosphorylated adenosine moiety of acetyl-CoA to SLC33A1 is stable, whereas the binding of the tail part, including pyrophosphate, pantothenic acid, β-mercaptoethylamine, and acetyl moieties, is relatively unstable (Fig. 1k, l; Supplementary Fig. S4 and Video S1). Notably, the pyrophosphate and pantothenic acid groups tend to shift towards TM10 (Fig. 1k; Supplementary Fig. S4). This finding reinforces our structural and functional data, demonstrating that the tail region of acetyl-CoA exhibits greater flexibility when binding to SLC33A1 (Fig. 1j; Supplementary Fig. S2b).

Interestingly, the mutation of aspartate residue Asp328 to alanine (D328A) led to a substantial increase in SLC33A1 activity (Fig. 1j). In our structural model, this residue is in close proximity to the phosphate moieties (5–6 Å) (Fig. 1h, i), and its mutation to alanine likely alleviates the electrostatic repulsion between the two negatively charged groups, thereby enhancing substrate binding and transport activity. This is corroborated by our MD simulation results (Supplementary Fig. S4d). It is worth noting that the crucial serine residue Ser113, whose mutation to arginine (S113R) is associated with the hereditary spastic paraplegia^8^, is also located in the vicinity of the acetyl-CoA-binding site (Fig. 1h). The substitution with an arginine residue introduces a bulky side chain that could potentially obstruct substrate binding or disrupt the local protein structure. Consistent with this hypothesis, our transport assays demonstrated a reduction in transport activity for the S113R mutation, but not for the S113A mutation (Fig. 1j), which aligns with previous research^9^.

Given that the 3′-phosphorylated ADP moiety of acetyl-CoA is essential for its interaction with SLC33A1 (Fig. 1h–j), we were interested in exploring whether other compounds containing a similar chemical group could also be recognized and transported by SLC33A1. We selected 5′-AMP, 3′-AMP, ADP, ATP, dATP, adenosine-3′-5′-diphophate, NADH, and NADPH for our analysis. The competitive [^3^H]-acetyl-CoA transport assay revealed that only ATP, dATP, and ADP could partially inhibit the transport activity of SLC33A1 when present at a 50-fold excess. However, the other compounds failed to suppress SLC33A1’s transport activity (Fig. 1m). These results suggest that while the 3′-phosphorylated ADP group is significant, the intact acetyl-CoA structure is necessary for its specific recognition and transport by SLC33A1.

To further understand the substrate transport cycle of SLC33A1, we predicted its structure in a lumen-facing conformation using AlphaFold3^14^. The modeled structure revealed that, similar to other MFS members, during the transition from the cytoplasm-facing to the lumen-facing conformation, the two halves of SLC33A1 close on the cytoplasmic side between TM4–5 and TM10–11, while opening on the luminal side between TM1–2 and TM7–8 (Fig. 1c–e; Supplementary Fig. S5a, b). Notably, in the lumen-facing conformation, SLC33A1 exhibits a wider lateral opening between TM2 and TM11, contrasting with the larger opening between TM5 and TM8 observed in the cytoplasm-facing conformation (Fig. 1c; Supplementary Fig. S5a). Importantly, in the lumen-facing state, the acetyl-CoA-binding site is disassembled and can no longer tightly associate with the substrate, as demonstrated by MD simulations (Supplementary Fig. S5c and Video S2). This finding aligns with the notion that, in this conformation, acetyl-CoA is readily released into the ER lumen.

Taken together, this study elucidates the molecular mechanisms underlying the substrate recognition by SLC33A1. It particularly highlights that the polar and π–π interactions between the 3′-phosphorylated ADP group of acetyl-CoA and SLC33A1 are paramount in determining their specificity. Moreover, the interactions between the remaining parts of the substrates and the protein also contribute, further refining their accurate identification (Fig. 1n). These insights not only advance our understanding of SLC33A1’s physiological functions but also hold significant potential to guide the drug development for the treatment of a range of diseases, including neurodegenerative disorders, aging-related conditions, and various forms of cancer^2,6^.

## Supplementary information

Supplementary Information

Supplementary Video S1

Supplementary Video S2

## Data Availability

Cryo-EM density map of the acetyl-CoA-bound SLC33A1 was deposited in the Electron Microscopy Data Bank under the accession code EMD-63562. The atomic coordinate has been deposited in the Protein Data Bank under the accession code 9M0S.
