# Supplementary material for: Mechanistic insights into the acetyl-CoA recognition by SLC33A1
Source: Cell Discov. 2025 Apr 10;11:36. doi: 10.1038/s41421-025-00793-1 (PMC11982209; doi:10.1038/s41421-025-00793-1)
Supplement: Supplementary file 1 — Supplementary Information [file 41421_2025_793_MOESM1_ESM.pdf]

Supplementary Information for

Mechanistic insights into the acetyl-CoA recognition by SLC33A1

Authors:

Dong Zhou[1,2], Nanhao Chen[2,3], Shitang Huang[4], Chen Song[2,3], and Zhe Zhang[1,2*]

Affiliations:

[1]State Key Laboratory of Membrane Biology, School of Life Sciences, Peking University, Beijing 100871, China

[2]Center for Life Sciences, Academy for Advanced Interdisciplinary Studies, Peking University, Beijing 100871, China

[3]Center for Quantitative Biology, Academy for Advanced Interdisciplinary Studies, Peking University, Beijing 100871, China

[4]Isotope Laboratory, School of Life Sciences, Peking University, Beijing 100871, China

[*]Correspondence should be addressed to zzhang01@pku.edu.cn.

The PDF file includes:

Methods

Supplementary References

Supplementary Figures S1-S5

Supplementary Table S1

Supplementary Videos S1-S2

Methods

Bacterial strains

SHuffle T7 Express Competent *E. coli* (New England Biolabs) cells were used to express Saposin A[1]. The transformed cells were grown in Terrific Broth (TB) medium until the optical density at 600 nm reached 0.8, and then induced with 0.5 mM IPTG at 18 °C overnight to induce protein expression. *E. coli* DH5a competent cells (TransGen Biotech) were used for molecular cloning and *E. coli* DH10Bac competent cells were used for production of recombinant bacmids, both of which were grown in Luria-Bertani (LB) medium with appropriate antibiotics.

Cell culture

HEK293S GnTI$^-$ cells (ATCC CRL-3022) were used for protein expression and maintained in Freestyle 293 Expression medium (Gibco) with shaking. The medium was enriched with 1% fetal bovine serum (FBS, VisTech). HEK293T cells were used for acetyl-CoA uptake assays and maintained in DMEM-high glucose medium (Cytiva) supplemented with 10% FBS and 1×penicillin–streptomycin. Mammalian cells were cultured at 37 °C with 5% CO_2. *Spodoptera frugiperda* (Sf9) cells (ATCC CRL-1711) were used to generate baculovirus and cultured in Sf-900 II SFM medium (Gibco) at 28 °C with agitation.

Expression and purification of Saposin A

Saposin A with an N-terminal 6×His tag and a PreScission protease cleavage site was expressed in *E.coli* Shuffle cells[2]. The cell pellets were lysed by sonication in Buffer A (50 mM HEPES pH 7.5, 300 mM NaCl, and 15% glycerol (v/v)). Cell debris was removed by centrifugation at 18,000 rpm for 1 h. Next, the supernatant was mixed with pre-equilibrated Ni-Smart beads (Smart-Lifesciences) and incubated at 4 °C for 1 h. The beads were then washed with 50 CVs of Buffer B (25 mM HEPES pH 7.25, 150 mM NaCl, and 5 mM imidazole). GST-tagged PreScission Protease was applied to remove the His-tag overnight. The protease was subsequently removed by binding to Glutathione beads. Further purification of the protein was achieved using a Superose 6 Increase 10/300 GL column equilibrated with Buffer C (25 mM HEPES pH 7.25 and 150 mM NaCl). The SaposinA-containing fractions were concentrated to 6 mg/ml and stored at –80 °C.

Expression and purification of human SLC33A1

The complementary DNA (cDNA) encoding human SLC33A1 was cloned into the pEG BacMam vector[3]. 10×His and green fluorescent protein (GFP) tags were attached to the C-terminus of SLC33A1 for affinity chromatography. Baculoviruses were produced by transfecting Sf9 cells with bacmids. After two rounds of amplification, viruses were used for mammalian cell transduction. When HEK293S GnTI$^-$ suspension cultures were grown to a density of 3×10^6 cells/ml at 37 °C, baculoviruses (10% v/v) were added to cultures and maintained for 12 h. Subsequently, 10 mM sodium butyrate was supplemented to the cultures, and the culture temperature was adjusted to 30 °C. Cells were harvested 48 h post-transduction.

The cell pellet expressing SLC33A1 was resuspended in Buffer A containing 2 μg/ml DNase I and protease inhibitor cocktail (APExBIO). The cells were then lysed with 2% n-dodecyl β-D-maltoside (DDM) and 0.2% Cholesteryl hemisuccinate (CHS) at 4 °C for 2 h. After centrifugation at 18,000 rpm for 30 min, the soluble fraction was incubated with Ni NTA Beads 6FF (Smart-Lifesciences) at 4 °C for 1 h. The resin was then washed with 50 column volumes (CVs) of Buffer D (25 mM HEPES pH 7.25, 150 mM NaCl, 0.02% DDM-0.002% CHS, and 20 mM imidazole) and 25 CVs of Buffer E (25 mM HEPES pH 7.25, 150 mM NaCl, 0.02% DDM-0.002% CHS, and 50 mM imidazole). SLC33A1 protein was eluted with 5 CVs of Buffer F (25 mM HEPES pH 7.25, 150 mM NaCl, 0.02% DDM-0.002% CHS, and 250 mM imidazole). The eluted protein was incubated with Saposin A[1] at a molar ratio of 1:15. After 1 h, Bio-Beads SM-2 Resin (BIO-RAD) was added to the sample to remove DDM-CHS and initialize the nanodisc reconstitution. After 14 h, Bio-Beads SM-2 Resin was added again to the protein mixture. After 5 h, the protein mixture was incubated with anti-GFP nanobody affinity resin at 4 °C for 1 h. The resin was then washed with 50 CVs of Buffer C. GST-tagged PreScission Protease was applied to remove the GFP-tag and release SLC33A1 from the resin. The protease was removed by binding to Glutathione beads (Smart-Lifesciences). The protein was further purified by size-exclusion chromatography (SEC) using a Superose 6 Increase 10/300 GL column (GE Healthcare) equilibrated with Buffer C. The peak fractions were concentrated with a 30 kDa cut-off centrifugal filter (Millipore) to around 10 mg/ml for cryo-EM experiments.

Cryo-EM sample preparation and data collection

For the SLC33A1/acetyl-CoA complex, the purified protein was incubated with 12 mM acetyl-CoA (Sigma) at 4 °C for 60 min, and 3 mM Fluorinated Fos-Choline-8 (Anatrace) was added prior to preparing the cryo-EM sample. A 3 µl protein sample was applied to glow-discharged holey carbon grids (Quantifoil R1.2/1.3 Au300). The grids were flash frozen in liquid ethane cooled by liquid nitrogen using a Vitrobot Mark IV (FEI) at 8 °C and 100% humidity. The blotting parameters were set at a blot time of 3 s and a wait time of 10 s. The grids were initially screened using a 200 kV Talos Arctica microscope (FEI) equipped with a Gatan K2 Summit detector. Raw movie stacks were recorded using a 300 kV Titan Krios microscope (FEI) with a K3 camera (Gatan) at a physical pixel size of 0.83 Å per pixel and a nominal defocus range of 1.0–2.0 µm. Each movie contained 40 frames. The total exposure dose was about 60 $e^{-}/Å^{2}$ and the exposure time was 3.2 s. The data collection parameters are summarized in Supplementary Table S1.

Cryo-EM image processing

The image stacks (7,500 micrographs) were gain normalized and corrected for beam-induced motion using Patch Motion Correction in cryoSPARC[4]. The CTF parameters were estimated using Patch CTF Estimation. The images with ice contamination or CTF values greater than 4, were discarded. An initial set of 200 micrographs were used for particle picking and generation of the 2D templates. Subsequently, particles were picked out using these 2D templates as reference by template picking and for training in the Topaz particle-picking

pipeline[5]. After two rounds of 3D classification, selected particles were used for Ab-initio reconstructions and heterogeneous refinements. Particles from the good classes were then combined, and duplicates were removed. Further iterative 3D classifications were conducted with subsequent Ab-initio reconstructions and heterogeneous refinements to eliminate suboptimal particles. Selected particles were then subjected to Non-Uniform (NU) refinement[6]. These particles served as seeds for the subsequent seed-facilitated guided heterogeneous refinement on the particles picked with a lower threshold[7]. Then, the low passed map was used for heterogeneous refinement, and the well-selected particles were further refined using NU refinement, yeilding a 3.5 Å-resolution map determined by the gold-standard FSC = 0.143 criterion. A detailed flowchart of the data process is presented in Supplementary Fig. S1.

Model building and refinement

The AlphaFold-predicted model of SLC33A1[8] was roughly fitted into the acetyl-CoA-bound SLC33A1 map using ChimeraX[9]. Then, real-space refinement was carried out using PHENIX[10], and manual adjustment was done in Coot[11]. Alternative automatic and manual refinements were performed before obtaining the final structural model. Local resolutions of the cryo-EM maps were estimated using cryoSPARC. Geometries of the structure models were validated by MolProbity[12]. All the structure related figures were generated using ChimeraX.

[^{3}H]-acetyl-CoA transport assay

Radioactive substrate transport assays were conducted using crude endoplasmic reticulum (ER) microsomes containing SLC33A1[13]. HEK293T cells transfected with wild-type (WT) or mutant SLC33A1 baculoviruses were cultured at 37 °C for 10-12 h, followed by the addition of 10 mM sodium butyrate and further cultured at 37 °C for an additional 16-20 h. The freshly harvested cells were washed twice with cold PBS and resuspended in 500 μl of cold ST buffer (25 mM Tris-HCl pH 7.4/4 °C, 300 mM sucrose, and protease inhibitor cocktail) to a density of approximately 3×10^7 cells/ml. The resuspended cells were lysed using a glass homogenizer[14]. Cell debris was removed by centrifugation at 1,000 g at 4 °C for 10 min, and the resulting supernatant was considered as crude ER microsome.

For the [^{3}H]-acetyl-CoA transport assay, 50 μl of the ER microsome suspension was mixed with 100 μl of transport buffer containing 25 mM Tris-HCl pH 7.4/4 °C, 300 mM sucrose, protease inhibitor cocktail, 5 μM unlabeled acetyl-CoA, and 9.6 pM [^{3}H]-acetyl-CoA (3.2 Ci/mmol, American Radiolabeled Chemicals, Inc.). After incubation for 5 min at 30 °C, the transport reaction was terminated by adding cold ST buffer and vacuum filtration through GF/C glass fibers filters (Whatman), followed by a 2-ml wash with ice-cold ST buffer. The radioactivity bound to the filters was measured by scintillation counting in 3 ml of scintillation solution (PerkinElmer, USA)[15].

The competition assay for [^{3}H]-acetyl-CoA transport by various small molecules was performed similarly, with the exception that the crude ER microsome suspension (50 μl) was incubated and reacted with 250 μM concentrations of the small molecules for 5 min at 37 °C.

To verify the protein expression level, crude ER microsomes were lysed with RAPI lysis buffer (25 mM Tris-HCl pH 7.5, 150 mM NaCl, 1 mM EDTA, 1% NP-40, 0.1% sodium dodecyl sulfate (SDS), and protease inhibitors) on ice for 30 min. After removing the cell debits by centrifugation at 15,000 rpm for 1 h, the supernatant was mixed with loading buffer and applied for SDS–PAGE analysis. The GFP fluorescence signal was detected using the GEL image system (Tanon). For normalization between WT and control, an equal number of cells were used for experiments (Supplementary Fig. S3).

Molecular dynamics (MD) simulations

The MD simulations for the cytoplasm-facing SLC33A1 were initiated from the cryo-EM structure, while those for the lumen-facing conformation were initiated from the AlphaFold3-predicted structure[16]. Among the 100 predicted structures, approximately 10 adopted the lumen-facing conformation. We randomly selected one for simulation, as they were all highly similar.

For the cytoplasm-facing SLC33A1 simulations, AlphaFold3 was first utilized to repair the non-terminal missing loops, namely residues 279-293 and residues 477-504, as shown in Supplementary Fig. S4a. Second, the pK_a values of the acidic and basic residues were calculated using the PROPKA3 program[17]. Three acidic residues that have the pK_a values higher than 7 were protonated: Glu187, Asp194, and Glu513. Next, the membrane-protein complex was set up using CHARMM-GUI[18,19]. The lipid ratio POPC:POPE:POPS was set to 55:30:15, with a total of 230 lipids (132 POPC, 66 POPE, and 32 POPS) used to construct the

bilayer membrane. The position of membrane bilayer was determined using the PPM web server[20]. In addition, about 22,000 TIP3P[21] water molecules were added around both sides of the bilayer membrane, and 34 sodium ions were inserted into the system to balance the total charge. Finally, the AMBER simulation input files were generated for subsequent MD simulations[22]. In the simulation system, the behaviors of all the lipids were described by the CHARMM all-atom additive forcefield[23], the protein was described by the CHARMM36m forcefield[24], and acetyl-CoA was depicted by the CHARMM General forcefield generated by CGenFF[25]. For the lumen-facing SLC33A1 simulations, a similar setup was employed, with the acetyl-CoA ligand positioned in the binding site by superimposing the cytoplasm-facing and lumen-facing protein structures.

The multi-step process of the conventional MD simulations was carried out to relax the entire system. Initially, a 5,000-step energy minimization was conducted with restraints on protein and ligand atoms (10 kcal/mol/Å^2) and the lipids atoms (2.5 kcal/mol/Å^2). Subsequently, a 150 ps heating process was taken to heat up the system to 300 K, followed by five-step equilibrium processes to equilibrate the density under 1 atm pressure. During these steps, restraints on the system were gradually decreased to zero. Finally, 300 ns MD simulations were carried out under the NPT ensemble. The Langevin thermostat algorithm, with a collision frequency of 1.0 ps^{-1}, was set to control the system temperature, while the Monte Carlo barostat was used to control the semi-isotropic pressure[26]. The cutoff value of the nonbonding interaction was set to 12 Å with a 10 Å switching-function value. The particle-mesh Ewald (PME) method was selected to described the long-range summation of the electrostatic

interactions, and bonds involving hydrogen were constrained using the SHAKE algorithm[27]. After the simulations, the binding free energies were calculated using the MM/GBSA algorithm[28]. All the simulations were performed using AMBER24[29].

Supplementary References

1. Pidathala, S. et al. Mechanisms of neurotransmitter transport and drug inhibition in human VMAT2. Nature 623, 1086-1092 (2023).
2. Frauenfeld, J. et al. A saposin-lipoprotein nanoparticle system for membrane proteins. Nat Methods 13, 345-351 (2016).
3. Goehring, A. et al. Screening and large-scale expression of membrane proteins in mammalian cells for structural studies. Nature Protocols 9, 2574-2585 (2014).
4. Punjani, A., Rubinstein, J. L., Fleet, D. J. & Brubaker, M. A. cryoSPARC: algorithms for rapid unsupervised cryo-EM structure determination. Nature Methods 14, 290-296 (2017).
5. Bepler, T. et al. Positive-unlabeled convolutional neural networks for particle picking in cryo-electron micrographs. Nature Methods 16, 1153-1160 (2019).
6. Punjani, A., Zhang, H. & Fleet, D. J. Non-uniform refinement: adaptive regularization improves single-particle cryo-EM reconstruction. Nature Methods 17, 1214-1221 (2020).
7. Wang, N. et al. Structural basis of human monocarboxylate transporter 1 inhibition by anti-cancer drug candidates. Cell 184, 370-383.e313 (2021).
8. Jumper, J. et al. Highly accurate protein structure prediction with AlphaFold. Nature 596, 583-589 (2021).
9. Goddard, T. D. et al. UCSF ChimeraX: Meeting modern challenges in visualization and analysis. Protein Sci 27, 14-25 (2018).

10. Afonine, P. V. et al. Real-space refinement in PHENIX for cryo-EM and crystallography. Acta Crystallogr D Struct Biol 74, 531-544 (2018).

11. Emsley, P., Lohkamp, B., Scott, W. G. & Cowtan, K. Features and development of Coot. Acta Crystallogr D Biol Crystallogr 66, 486-501 (2010).

12. Davis, I. W. et al. MolProbity: all-atom contacts and structure validation for proteins and nucleic acids. Nucleic Acids Res 35, W375-383 (2007).

13. Jonas, M. C., Pehar, M. & Puglielli, L. AT-1 is the ER membrane acetyl-CoA transporter and is essential for cell viability. Journal of Cell Science 123, 3378-3388 (2010).

14. Cox, B. & Emili, A. Tissue subcellular fractionation and protein extraction for use in mass-spectrometry-based proteomics. Nature Protocols 1, 1872-1878 (2006).

15. Zhang, Y. et al. Structural insights into VAChT neurotransmitter recognition and inhibition. Cell Research 34, 665-668 (2024).

16. Abramson, J. et al. Accurate structure prediction of biomolecular interactions with AlphaFold 3. Nature 630, 493-500 (2024).

17. Olsson, M. H. M., Sondergaard, C. R., Rostkowski, M. & Jensen, J. H. PROPKA3: Consistent Treatment of Internal and Surface Residues in Empirical Predictions. J Chem Theory Comput 7, 525-537 (2011).

18. Jo, S., Klauda, J. B. & Im, W. CHARMM-GUI Membrane Builder for Mixed Bilayers and Its Application to Yeast Membranes. Biophys J 96, 41a-41a (2009).

19. Park, S., Choi, Y. K., Kim, S., Lee, J. & Im, W. CHARMM-GUI Membrane Builder for Lipid Nanoparticles with Ionizable Cationic Lipids and PEGylated Lipids. J Chem Inf Model 61, 5192-5202 (2021).

20. Lomize, M. A., Pogozheva, I. D., Joo, H., Mosberg, H. I. & Lomize, A. L. OPM database and PPM web server: resources for positioning of proteins in membranes. Nucleic Acids Res 40, D370-D376 (2012).

21. Jorgensen, W. L., Chandrasekhar, J., Madura, J. D., Impey, R. W. & Klein, M. L. Comparison of Simple Potential Functions for Simulating Liquid Water. J Chem Phys 79, 926-935 (1983).

22. Lee, J. et al. CHARMM-GUI supports the Amber force fields. J Chem Phys 153 (2020).

23. Venable, R. M. et al. CHARMM All-Atom Additive Force Field for Sphingomyelin: Elucidation of Hydrogen Bonding and of Positive Curvature. Biophys J 107, 134-145 (2014).

24. Huang, J. et al. CHARMM36m: an improved force field for folded and intrinsically disordered proteins. Nat Methods 14, 71-73 (2017).

25. Vanommeslaeghe, K. et al. CHARMM General Force Field: A Force Field for Drug-Like Molecules Compatible with the CHARMM All-Atom Additive Biological Force Fields. J Comput Chem 31, 671-690 (2010).

26. Bernetti, M. & Bussi, G. Pressure control using stochastic cell rescaling. J Chem Phys 153, 114107 (2020).

27. Macuglia, D. SHAKE and the exact constraint satisfaction of the dynamics of semi-rigid molecules in Cartesian coordinates, 1973-1977. Arch Hist Exact Sci 77, 345-371 (2023).

28. Wang, E. C. et al. End-Point Binding Free Energy Calculation with MM/PBSA and MM/GBSA: Strategies and Applications in Drug Design. Chem Rev 119, 9478-9508 (2019).

29. AMBER 2024 (University of California, San Francisco, 2024).

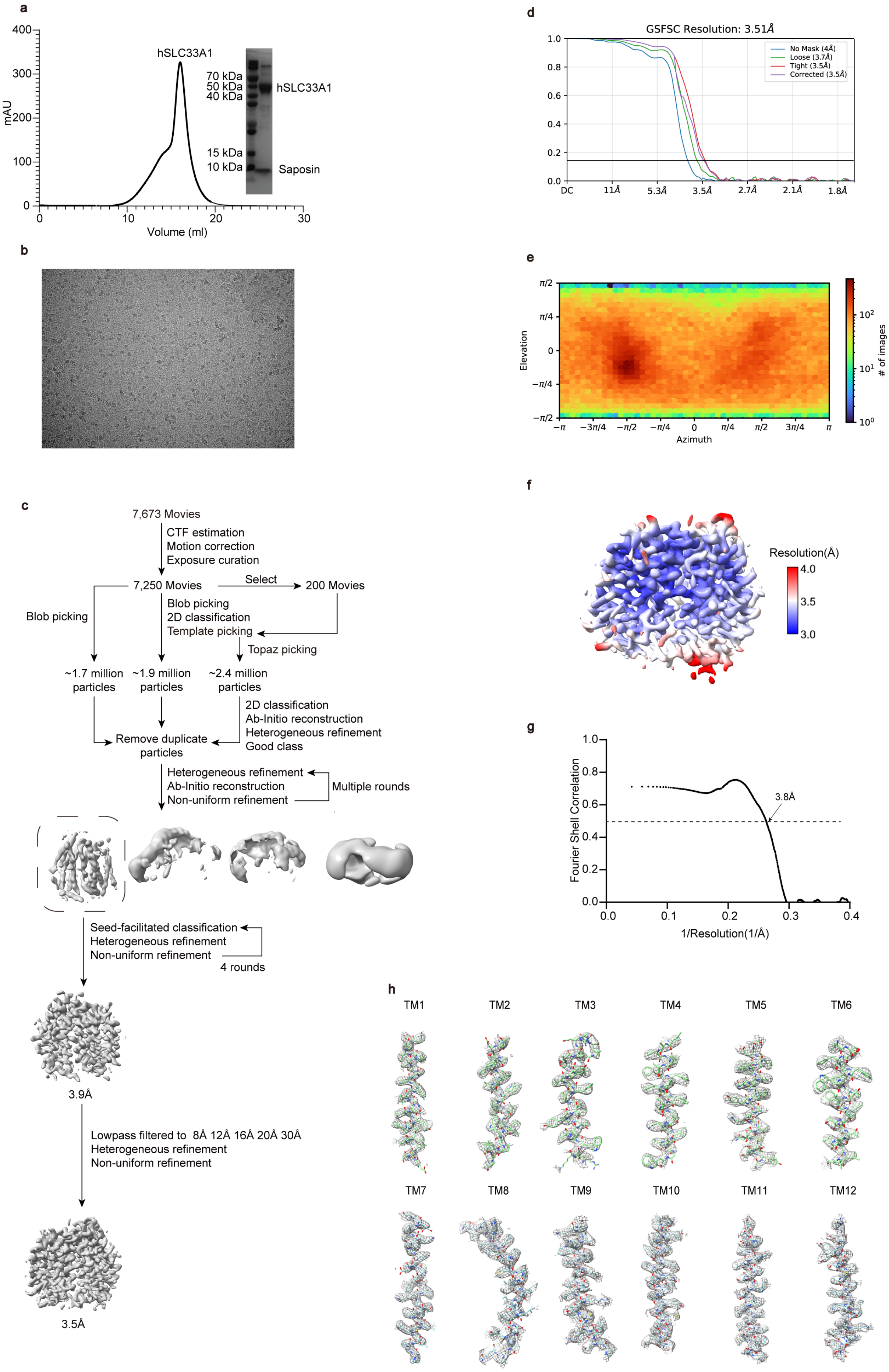
a
hSLC33A1
70 kDa
50 kDa
40 kDa
hSLC33A1
15 kDa
10 kDa
Saposin
mAU
Volume (ml)
b
c
7,673 Movies
CTF estimation
Motion correction
Exposure curation
7,250 Movies
Select
200 Movies
Blob picking
Blob picking
2D classification
Template picking
Topaz picking
~1.7 million particles
~1.9 million particles
~2.4 million particles
2D classification
Ab-Initio reconstruction
Heterogeneous refinement
Good class
Remove duplicate particles
Heterogeneous refinement
Ab-Initio reconstruction
Non-uniform refinement
Multiple rounds
Seed-facilitated classification
Heterogeneous refinement
Non-uniform refinement
4 rounds
3.9Å
Lowpass filtered to 8Å 12Å 16Å 20Å 30Å
Heterogeneous refinement
Non-uniform refinement
3.5Å
d
GSFSC Resolution: 3.51Å
No Mask (4Å)
Loose (3.7Å)
Tight (3.5Å)
Corrected (3.5Å)
DC
11Å
5.3Å
3.5Å
2.7Å
2.1Å
1.8Å
e
Elevation
Azimuth
# of images
f
Resolution(Å)
4.0
3.5
3.0
g
Fourier Shell Correlation
3.8Å
1/Resolution(1/Å)
h
TM1
TM2
TM3
TM4
TM5
TM6
TM7
TM8
TM9
TM10
TM11
TM12

Fig. S1 Cryo-EM data processing of the acetyl-CoA-bound SLC33A1 dataset.

a Profile of the size exclusion chromatography (SEC) and SDS–PAGE results. **b** Representative micrograph of particles. **c** Summary of the data processing procedures for SLC33A1 in complex with acetyl-CoA, all conducted using cryoSPARC. **d** Fourier shell correlation (FSC) curves between the two half maps. **e** Angular distribution of particles for the final 3D reconstruction. **f** Local resolution assessment of the cryo-EM map. **g** FSC curve calculated between the cryo-EM map and structural model. **h** Cryo-EM densities of the transmembrane helices.

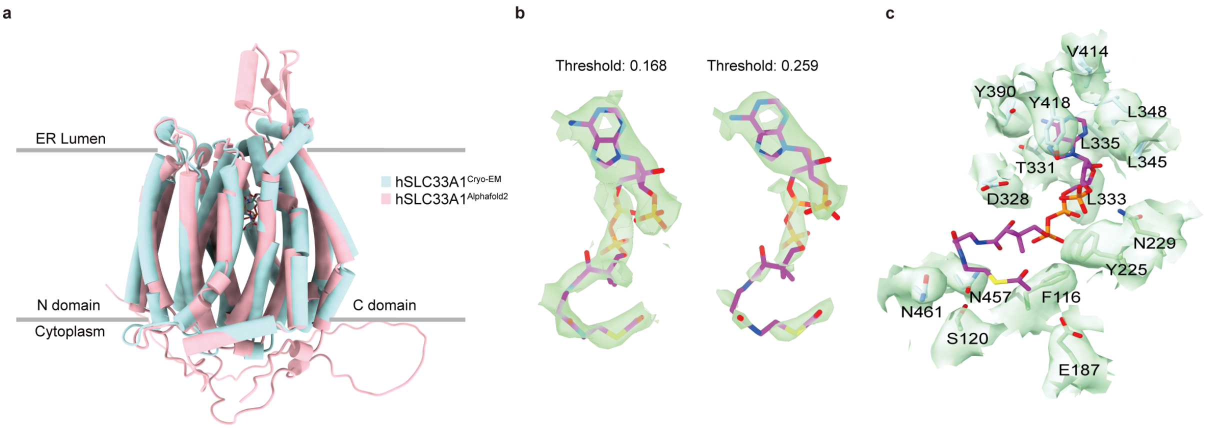

Fig. S2 Cryo-EM structure of the acetyl-CoA-bound SLC33A1.

a Superimposition of the apo-SLC33A1 structure predicted by Alphafold2 (PDB ID: AF-O000400-F1-v4) and the acetyl-CoA-bound SLC33A1 structure presented in this study. The root-mean-square deviation (RMSD) between the two structures is 1.5 Å. **b** Cryo-EM densities of acetyl-CoA at different thresholds. **c** Cryo-EM densities for the residues involved in acetyl-CoA binding.

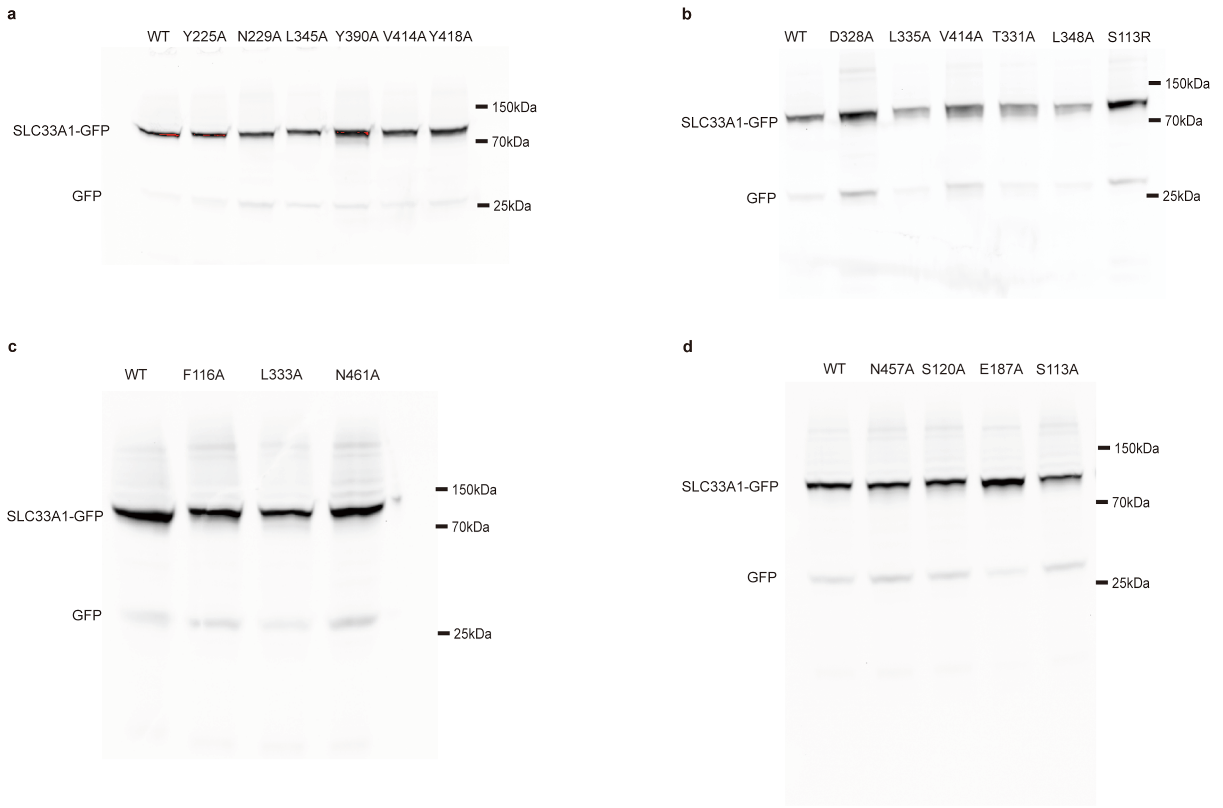

Fig. S3 Detection of the SLC19A3 expression by SDS–PAGE.

a-d Verification of the expression level of different SLC33A1 mutants by GFP signal. All experiments were repeated three times with similar results.

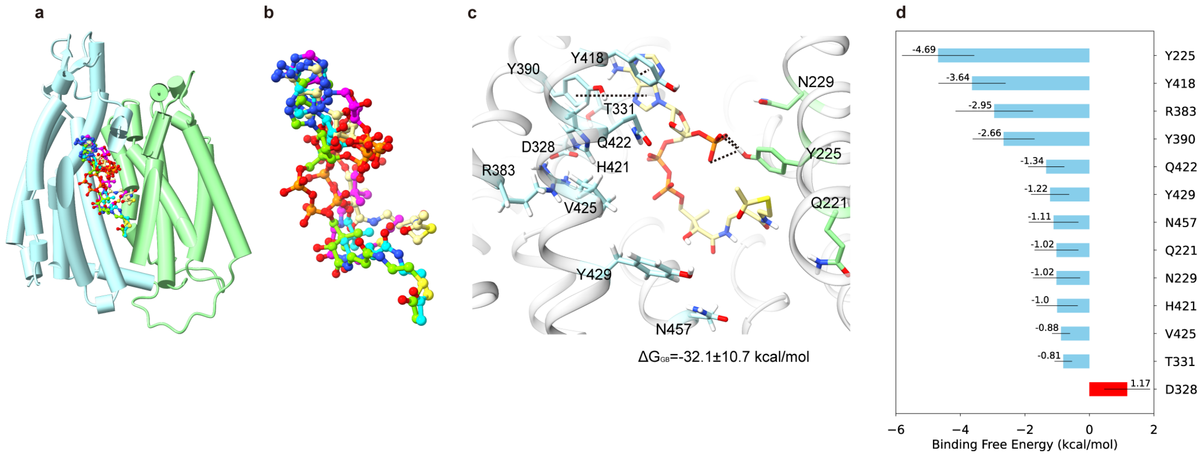

Fig. S4 The stable substrate binding models of SLC33A1 calculated using MD simulations.

a The stable binding models of acetyl-CoA within SLC33A1 during the 300 ns MD simulation. **b** Comparison of acetyl-CoA poses at various time points during the 300 ns MD simulation. The colors of acetyl-CoA in (**a**) and (**b**) correspond to the snapshots shown in Fig. 1k. **c, d** The interaction details between acetyl-CoA and SLC33A1 at the 300 ns MD simulation. Key residues contributing to acetyl-CoA binding are indicated in (**c**), and their respective free energy contributions are detailed in (**d**).

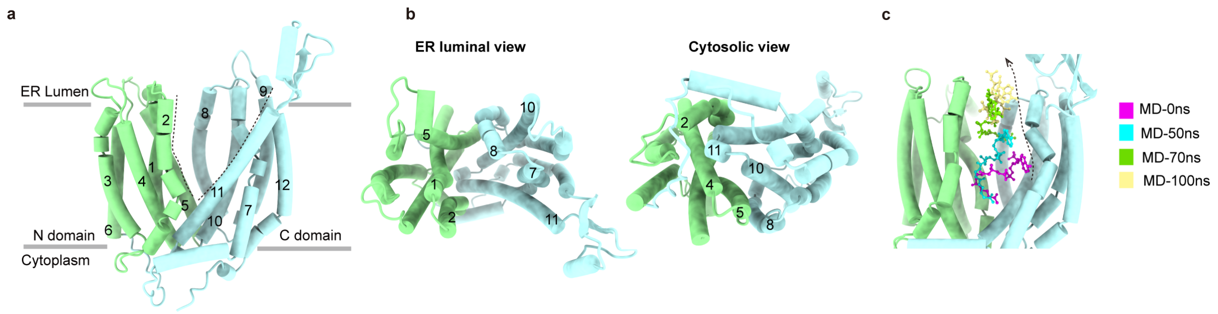

Fig. S5 Structure and dynamics of SLC33A1 in the lumen-facing conformation.

a, b AlphaFold3-predicted structure of SLC33A1 in the lumen-facing conformation, viewed from the membrane plane **(a)**, the ER luminal side **(b,** left**)**, or the cytosolic side **(b**, right**)**. TMs 1-6 (N domain) are colored in light green, while TMs 7-12 (C domain) are shown in pale turquoise. **c** Comparison of acetyl-CoA poses at various time points during the 100 ns MD simulations. The colors of acetyl-CoA correspond to snapshots of its release into the ER lumen. SLC33A1 is represented in a cylinder model, while acetyl-CoA is shown in a stick-ball representation.

Table S1 Cryo-EM data collection, refinement, and validation statistics.

	Acetyl-CoA-bound SLC33A1 (EMD-63562) (PDB 9M0S)
**Data collection and processing**	
Magnification	105,000
Voltage (kV)	300
Electron exposure (e^{-}/Å^2)	60
Defocus range (μm)	1.0-2.0
Pixel size (Å)	0.83
Symmetry imposed	C1
Initial particle images (no.)	6,537,482
Final particle images (no.)	238,758
Map resolution (Å)	3.5
FSC threshold	0.143
Map resolution range (Å)	3.0-5.0
**Refinement**	
Model resolution (Å)	3.8
FSC threshold	0.5
Map sharpening *B* factor (Å^2)	-204.5
Model composition	
Non-hydrogen atoms	3,413
Protein residues	424
Ligands	1 (Acetyl-CoA)
B factors (Å^2)	
Protein	81.16
Ligand	88.10
R.m.s. deviations	
Bond lengths (Å)	0.007
Bond angles (°)	1.090
Validation	
MolProbity score	1.52
Clashscore	5.66
Poor rotamers (%)	0.00
Ramachandran plot	
Favored (%)	96.65
Allowed (%)	3.35
Disallowed (%)	0.00

Video S1 300-ns MD simulations of acetyl-CoA binding modes in the cytoplasm-facing conformation of SLC33A1.

Video S2 120-ns MD simulations of the acetyl-CoA binding modes in the AlphaFold3-predicted lumen-facing conformation of SLC33A1.
